# Correction: Spatial distribution of animal source food consumption and associated factors among children aged 6–23 months in Ethiopia: A geographically weighted regression analysis

**DOI:** 10.1371/journal.pone.0332507

**Published:** 2025-09-15

**Authors:** Mekuriaw Nibret Aweke, Amare Mesfin, Gebrie Getu Alemu, Berhanu Mengistu, Tewodros Getaneh Alemu, Habtamu Wagnew Abuhay

The fourth author’s name is spelled incorrectly. The correct name is: Berhanu Mengistu.
