# Supplementary material for: Spatial distribution of animal source food consumption and associated factors among children aged 6–23 months in Ethiopia: A geographically weighted regression analysis
Source: PLoS One. 2025 May 20;20(5):e0324855. doi: 10.1371/journal.pone.0324855 (PMC12092019; doi:10.1371/journal.pone.0324855)
Supplement: Attachment — Submitted filename: Response to reviewers .docx [file pone.0324855.s002.docx]

**PLOS ONE Editorial Office	Date 28 April, 2025**

**Dear Editor,**

**Subject: Resubmission of Revised Manuscript PONE-D-24-34120**

I am pleased to resubmit the revised version of our manuscript titled " Spatial Distribution of Animal Source Food Consumption and Associated Factors Among Children Aged 6–23 Months in Ethiopia: A Geographically Weighted Regression Analysis" (Manuscript ID: PONE-D-24-34120). We have carefully addressed the comments and suggestions provided by the reviewers and editorial team.

In the accompanying response document, we have detailed how each point was addressed, and we have made appropriate revisions to the manuscript. We believe these changes have improved the quality of our work, and we are hopeful that the revised version now meets PLOS ONE's publication standards.

Thank you for the opportunity to revise and resubmit our manuscript. We look forward to your feedback and hope for a favorable decision.

**Sincerely,**

**Mekuriaw Nibret Aweke**

**Correspondent Author**

**Point by point response to the Academic editor and reviewer’s comments**

| 1. **Comments from the academic editor and responses** | |
| --- | --- |
| **Academic editor’s comments** | **Authors’ responses** |
| Abstract and Conclusion: Clarify the main contributions and practical implications of the study. | **Dear editor,**  Thank you for your feedback. We have corrected the abstract and conclusion by clarifying the main contributions and practical implications of the study |
| Introduction: Improve the logical flow of paragraphs and refine redundant statements. | **Dear editor,**  We appreciate your feedback regarding the logical flow of paragraphs and to refine redundant statements. We have carefully reviewed the sections and revise each paragraph and solve these issues. |
| **Justification and Methods**: Strengthen the rationale for the study focus and analytical approach; clarify data preprocessing and variable treatment; ensure consistent notation and define all abbreviations at first mention. | **Dear editor,**  Thank you for your suggestion to strengthen the rational for the study and analytical approach. We have revised the rational of the study by making clear and precise and we also revised the methodologies section based on your suggestions carefully. |
| **Results and Figures**: Correct table percentages, improve figure references and readability, and standardize formatting and terminology. | **Dear editor,**  Thank you for your recommendation to correct table percentages, improve figure references and readability, and standardize formatting and terminology. We have corrected the table percentage for better readability. we revise each figure reference carfuly for better readability by standardizing the formatting and terminology.  Thank you again for your suggestions. |
| Address study limitations (e.g., data source), expand contextual discussion (e.g., existing programs and socioeconomic aspects), and streamline content by removing redundant sections. | **Dear Editor,**  Thank you for your feedback. We have adress the study limitations and we revise the discussion section by expanding this section by triangulating with existing programs and socio economic status. We also rephrase and correct the logical flow of stetments by removing redundant sections. |
| Recommendations: Provide more specific, actionable recommendations based on the findings. | **Dear editor, thank you for your recommendations to provide specific and actionable recommendations based on our findings. We have revised the recommendation based on your suggestions. Thank you again for your help to this much for better enhancement of the manuscripit.** |
| Please ensure that your manuscript meets PLOS ONE's style requirements, including those for file naming. | **Dear editor,**  Thank you for the opportunity to submit our manuscript.  We have corrected the document to fulfil plose one publication requirements. |
| Please confirm at this time whether or not your submission contains all raw data required to replicate the results of your study. | Dear editor, thank for your enquiry to confirm the data availability stetement. All required data are found in the manuscript and the orginal data is found in DHS measure and can be access by request from the DHS measure. |
| We note that Figures 4, 5, 6, 7, 9, 10, 11, 12 and 13 in your submission contain [map/satellite] images which may be copyrighted. | Dear Editor,  Thank you for your inquiry regarding the figure sharing conditions. We confirm that the map images in Figures 4, 5, 6, 7, 9, 10, 11, 12 and 13 were generated using shapefiles from the Central Statistical Agency (CSA) of Ethiopia, available on Open Africa Data at https://africaopendata.org/dataset/ethiopia-shapefiles. These shapefiles are licensed under CC BY 4.0, allowing sharing and distribution with proper attribution. We have updated the figure captions to include the source and ensure compliance with copyright requirements. |
| **2. Comments from reviewer #1 and responses** | |
| **Reviewer’s comments** | **Authors’ responses** |
| The paper addresses the topic of child nutrition and utilizes a geospatial technique that is increasingly used in public health. It shows originality and makes a significant contribution to the topic. The empirical strategies are well explained. However, there are a few areas that could be revised to enhance the content of the manuscript. | Dear reviewer, thank you for your valuable feedback. we appreciate your recognition of the originality and contribution of the paper, as well as your positive comments. We have revised the contents of the manuscript based on your comments and suggestions. |
| 1. Conclusion in the abstract should be redrafted to highlight the contribution of the article and/or specific implication for practice or methodology. | Dear reviewer,  Thank you for your feedback. We have revised the conclusion in the abstract to highlight the contributions of our article. |
| A strong rationale for focusing on animal food source consumption of children aged 6-23 months is missing. Additionally, consider adding some strong justification for why the authors employed a geographically weighted regression technique to identify associated factors. | Dear reviewer,  Thank you for your suggestion and comments. We have revised by incorporating strong rational for focusing on animal food source consumption of children aged 6-23 months in the introductions section. We have added strong justifications to employed a geographically weighted regression technique to identify associated factors in the introduction section and in the methodology sections. Thank you very much for your valuable suggestions. |
| Please write acronyms/abbreviations in the long term when used for the first time. There are places where the abbreviation is used before it is introduced. | **Dear reviewer,**  Thank you for your feedback. We have corrected this issue by introducing the abrivation and acronyms when first use.  Thank you again for your valuable comments. |
| A clear explanation of the data preprocessing is required. E.g. how do you handle missing values in the dependent variable and independent variables? | **Dear Reviewer,**  Thank you for your feedback. We have revised the data management and analysis section by explaining the data processing including managing missing values as the following.  “*Records with missing values in the outcome variable (ASF consumption) were excluded, as per DHS recommendations to retain only cases with complete outcome information. For independent variables, a complete case analysis was used since the proportion of missing data was small. Sampling weights (v005/1,000,000) were applied to adjust for the multi-stage sampling design and ensure representativeness, following DHS guidelines*”  Thank you again for your unreserved comment and suggestions, |
| Percentages in Table One should be revised and need to be calculated properly. | **Dear reviewer,**  Thank you for your feedback. We have revised the table percantage and we recalculated the frequency for better clarity and readabilty. We understand that the former table was not clrear for readers. Thank you again for your helpful comments.. |
| The spatial analysis was done at the regional level. I think it would be better to do it at the sub-national level (preferably at the Zonal or woreda level). | **Dear reviewer,**  Thank you for your suggestion. Due to the large number of zones and woredas, we prefered to conduct the analysis at the regional level to ensured clearer presentation and more reliable results. However, we are open to revisiting the analysis at the zonal or woreda level. Thank you again for your relevance comments. |
| More appropriate recommendations and precise comparisons of determinants in this study relative to existing literature, e.g., (such as religion were found to be significant in this study), are expected to aid in the understanding and interest of readers on this important topic. | **Dear reviewer,**  Thank you for your feedback.. we have revised the recommendations and we also add more d comparisons with the existing literature in the discussion section. |
| It can be noted that the dependent variable of ASF came from self-report and this would undermine validity and reliability. I would like to ask the authors to address this issue in the section of the Discussion. | **Dear reviewer,**  Thank you for your valuable feedback. We acknowledge the concern regarding the use of self-reported data for the dependent variable of animal source food (ASF) consumption, which could potentially affect the validity and reliability of the findings. We have addressed this issue in the discussion section by acknowledging the limitations of self-reporting, such as recall bias under strength and limitations of the study as the following:  “---*However, this study has a few limitations. The data from the Mini-EDHS 2019 are cross-sectional, so causality cannot be established. Additionally, self-reported dietary data may introduce recall bias….”*  **Thank you for your insightful comments and valuable suggestions** |
| **3. Comments from reviewer #2 and responses** | |
| The article is interesting and well structured. The rationale is justified, and the methodology is solid and adequate…. | **Dear reviewer,**  Thank you very much for your appreciations and positive feedback to this work. |
| **Abstract: Put the acronym ASF adjacent to "Animal source foods"** | **Dear reviewer,** Thank you for your valuable comments. We have put the acronym ASF adjacent to "Animal source foods" which will make the abstract easier to read. |
| Check the writing of the variables, there are differences between the equation and the text. For example, the equation presents two variables as "Bk (ui, vi)" and "Ei", while they are written in the text as "βk (ui, vi)" and "εii". | **Dear reviewer,**  Thank you for your comments. We have corrected the variable difference in the equation and the text under the geographically weighted regression analysis section. The correct one is “εi” and we have corrected it in both the equation and in the text. Thank you again for your concern to this much for better improvement of the work. |
| Have you tested a possible correlation between the sociodemographic characteristics and the socioeconomic factors considered (wealth, education and children per household)? | **Dear reviewer.**  Thank you for your inquiry. The possible correlation between the sociodemographic characteristics and the socioeconomic factors were adressed. And we have explained it under the methodology section as the following:  **“***The final model was validated through internal cross-validation, and we checked for multicollinearity (with a variance inflation factor of less than 7.5) to ensure that the independent variables were not redundant***”** |
| In the "Spatial autocorrelation by distance" graph (Figure 3), please put the distance in kilometers instead of meters. | **Dear reviewer,**  Thank you for your valuable suggestion. We have put the distance in kilometers. We believe this graph is now easy to understand. |
| In the maps (Figures 4 to 7 and 9 to 13), some areas’ names are difficult to read. Maybe try to adjust the points’ or polygons’ (generated by the analysis, not the base polygon with the areas) opacity to make it more transparent. | **Dear reviewer,**  Thank you for your suggestions. Some areas names are difficult to read this is due to the small radius in that area and specifically two areas close together make it more difficult to read. We make the names label more readable and we hope this make the areas name more readable. Thank you for your suggestions to improve the map quality and enhance the overall document quality. |
| In Figure 4, change the color of the "non significant" results to make it different from the "Hot Spots". In Figure 5, for instance, this issue is nonexistent. | **Dear reviewer,**  Thank you for your comment. We have changed the color of the "non-significant" results to make it different from the "Hot Spots" and now we hope it is clear to read. Thank you again for your valuable suggesions |
| In Figure 7, change the legend title from "Most likly clusters" to "Most likely clusters". | Dear reviewer ,  Thank you for your reccomendation. we apologize to the spelling error that we made. We have changed legend title from "Most likly clusters" to "Most likely clusters". |
| In Figure 11 ("figure 11.tif"), review the color palette because the first two categories ("-0.272 to -0.223" and "-0.223 to -0.169") have similar colors. | Dear reviewer ,  Thank you for your reccomendation. we have corrected the color palette because the first two categories ("-0.272 to -0.223" and "-0.223 to -0.169") have similar colors. |
| Please check the reference to the figures from Figure 9 to 13 in the text. The "Figure 9" in the text appears to be referring to Figure 10 ("Figure he 10.tif"), while “Figure 10” is referring to Figure 11 ("figure 11.tif"), etc. And I did not understand to which part of the text the "figure 9 sec.tif" file is related. | Dear reviewer ,  Thank you for your comment. We have reviewed and corrected the figure references from Figures 9 to 13 in the text to match the correct figure files. Additionally, we have clarified the placement of "figure 9 sec.tif" to ensure it aligns properly with the corresponding part of the text. |
| 4. **Comments from reviewer #3 and responses** | |
| The article is well-written and presents a strong line of reasoning based on the literature…. | Dear reviewer ,  Thank you for your positive feedback for this work. We appreciate your constrictive and interesting comment to the manuscript |
| Title: Appropriate and effectively conveys the study’s purpose | Dear reviewer ,  Thank you very much for you understanding to appropriateness to the title. |
| The introduction presents and connects relevant facts related to the theme, justifying the need for the study. However, reorganizing the paragraphs could improve the flow and clarity of the argument | Dear reviewer ,  Thank you for your comment and suggestions. We have revised the introduction section by correcting and reorganizing paragraphs and stetments to improve the clarity of argument. we have reorganized the paragraphs and stetments by fisrt introducing about malnutrition and the we mentioned about proper nutriton for young children by focusing on animal source food consumptions. And we continue the introductions about the significance of animal source food consumption for young child health groth and development. We also include the current animal source food consumptions status and we also state factors affecting for low consumption of animal source food and the current implemented programs and strategies to increase animal source food consumption to reduce the burdens of undernutriton, We hope now the introduction has good logical flow and clear arguments. |
| Additionally, revising the final sentence of paragraph 3 would help avoid repetition | Dear reviewer ,  Thank you for your comment and suggestions. We have revised the whole introduction section and now the repetitions of ideas are corrected |
| Methods: The methods section is well-structured and provides a detailed description of the strategies employed, especially regarding statistical procedures… | Dear reviewer ,  Thank you for your positive feedback to the methods section and its relevance. |
| Results: The results are well-organized and clearly presented, effectively supporting the subsequent discussion. | Dear Reviewer,  We are pleased to receive your positive feedback regarding the results section, which you found well-organized and clearly presented, effectively supporting the subsequent discussion. |
| A careful review of the figure numbering is recommended to ensure alignment with the order in which they are referenced in the text. In the reviewed file, some figures appeared out of sequence, which could lead to confusion for the reader. | Dear Reviewer,  Thank you for your valuable comments and suggestions. Regarding the sequence of the figures, we noticed that there was a discrepancy between the figure order in the text and the figure references. We have now corrected this issue. We appreciate your helpful input, which has contributed to enhancing the quality of this work. |
| Additionally, improving the visual quality of the figures would enhance the graphical representation of the data | Dear Reviewer,  Thank you for your helpful comment regarding the quality of the figure. We have reanalyzed the figure using ArcMap, and some figures are now visible. However, after exporting from ArcMap and converting the files to TIFF format, we noticed that the quality of the figure was still reduced. We remain open to any further suggestions for improving the figure quality if it is still not up to standard. |
| Discussion: The discussion thoroughly addresses all findings, contextualizing them within existing literature, which reinforces theoretical grounding and consistency. | Dear reviewer ,  We are pleased to receive your positive feedback regarding the discussion section, particularly regarding its strengths and writing style |
| However, the repetitive structure of the paragraphs—each following a similar format—makes the reading somewhat monotonous. A rewrite is suggested to improve fluency and dynamism, creating a more engaging and cohesive reading experience. | Dear reviewer ,  Thank you for your recommendation to improve the flow of ideas and to correct the paragraph formats since they are similar that makes the reading monotonous. We have revised the discusion section and correct the flow of ideas and correct the format for better idea flow and to increase its readability. |
| Furthermore, the authors briefly mention the existence of nutritional programs in Ethiopia but do not provide details or references | Dear reviewer,  Thank you for your valuable comment and suggestions regarding to add references about the current Ethiopian nutritional p rograms.we have added the reference by stating with the current main nutriton strategies and programs implmented in Ethiopia as the following  “*Ethiopia has adopted the Food-Based Dietary Guidelines, the National Nutrition Program, the Food and Nutrition Policy, and the National Food and Nutrition Strategy as comprehensive measures to improve dietary practices, combat undernutrition, and promote the consumption of animal source foods(32, 33)*.” |
| Similarly, it would be valuable to elaborate on socioeconomic factors, particularly household size and its potential influence on food acquisition. Incorporating national surveys or studies that collect this type of data could offer relevant insights into food insecurity and dietary intake in the studied context. | Dear Reviewer,  We appreciate your suggestion to enhance the discussion section by appropriately elaborating on the effect of large family size on food acquisition. we have revised this paragraph and included the relevant references. Thank you once again for your valuable assistance in improving the manuscript. |
| removing the last paragraph of the discussion is recommended, as it reiterates points already covered earlier in the section and in the introduction. Its omission would contribute to a more concise and focused discussion. | Dear reviewer,  Thank you for your suggestion. We have removed the last paragraph of the discusion section. |
| The section on limitations and strengths is excellent, reinforcing the methodological rigor applied throughout the manuscript. | Dear reviewer,  We are glad to hear this positive comment from you. |
| Conclusion: The conclusion effectively addresses the study’s objective, summarizing key findings clearly. However, the recommendations based on the results could be made more explicit. What existing policies or programs aimed at improving child nutrition in Ethiopia could be refined in light of the findings? Additionally, it would be beneficial to discuss the essential elements a new public policy should incorporate to promote increased consumption of animal-source foods among children, considering relevant social, economic, and cultural challenges | Dear reviewer.  We have revised the recommendation section by incorporation the new strategies and specific interventions based on the study finding and with clear and specific recommendations.  **Thank you agin for your unreserved feedback to improve the quality of this work.** |
